# Knowledge and perception of Prevention of Mother to Child services amongst pregnant women accessing antenatal clinic in a Primary Health Care centre in Nigeria

**DOI:** 10.4102/phcfm.v4i1.432

**Published:** 2012-10-30

**Authors:** Eme T. Owoaje, Adedoyin D. Omidokun, Olusimbo K. Ige

**Affiliations:** 1 Department of Community Medicine, University of Ibadan, Nigeria; 2 Malaria Action Program for States, United States Agency for International Development, Nigeria

## Abstract

**Background:**

Few studies have assessed pregnant women's perceptions regarding prevention of mother to child of HIV and the available services at the primary health care level in Nigeria.

**Objective:**

Assessment of knowledge and perception of antenatal clinic (ANC) attendees regarding Prevention of Mother to Child Transmission (PMTCT) of HIV at primary health care facilities in south-west Nigeria.

**Method:**

A cross-sectional survey was conducted amongst 400 antenatal attendees in a Primary Health Care centre in Ibadan, Nigeria.

**Results:**

Known methods of PMTCT were: use of anti-retroviral treatment (ART) during pregnancy (75.0%), ART at birth (65.8%) and not breastfeeding (61.8%). Previous HIV Counselling and Testing (HCT) was reported by 71%, significantly higher proportions of those who were married, in the third trimester of pregnancy or engaged in professional and/or skilled occupations had been tested. Regarding the HCT services provided, 92.2% understood the HIV-related health education provided, 89.7.2% reported that the timing was appropriate, 92.6% assessed the nurses’ approach as acceptable but 34.0% felt the test was forced upon them. Majority (79.6%) were aware of non-breastfeeding options of infant feeding, but only 3.5% were aware of exclusive breastfeeding for a stipulated period as an infant feeding option. Nevertheless, the majority of the women found the non-breast feeding option culturally unacceptable.

**Conclusion:**

Women in this survey were knowledgeable about the methods of PMTCT, but had negative perceptions regarding certain aspects of the HCT services and the recommended non-breastfeeding infant feeding option. Health workers should provide client friendly services and infant feeding counselling that is based on current WHO recommendations and culturally acceptable.

## Abstrait

**Connaissance et perception des services de prévention de la transmission de la mère à l'enfant chez des femmes enceintes bénéficiant de consultations prénatales dans un centre de soins primaires au Nigéria**

**Contexte:**

Peu d’études ont évalué la perception des femmes enceintes concernant la Prévention de la Transmission de la Mère à l'Enfant (PTME) du VIH et les services disponibles au niveau des soins de santé primaires au Nigéria.

**Objectif:**

L’évaluation de la connaissance et de la perception des femmes dans les cliniques prénatales concernant la PTME du VIH dans les centres de soins de santé primaires dans le sud-ouest du Nigéria.

**Méthode:**

Une enquête transversale a été réalisée auprès de 400 femmes bénéficiant de soins prénatals dans un centre de soins de santé primaires à Idaban, Nigéria.

**Résultats:**

Les méthodes de PTME connues sont: le traitement ART pendant la grossesse (75.0%), le traitement antirétroviral (ART) à la naissance (65.8%) et le fait de ne pas allaiter (61.8%). Un suivi antérieur de services de conseil et de dépistage du VIH a été déclaré par 71% des femmes, une proportion nettement plus élevée des femmes mariées, dans leur troisième trimestre de grossesse ou exerçant une activité professionnelle et/ou qualifiée ayant été dépistées. En ce qui concerne les services de conseil et de dépistage fournis, 92.2% ont compris l’éducation à la santé liée au VIH dispensée, 89.7% ont déclaré que le calendrier était approprié, 92.6% ont évalué l'approche des infirmières comme étant acceptable mais 34.0% ont considéré que le dépistage leur avait été imposé. La majorité (79.6%) connaissait les options d'alimentation du nourrisson sans allaitement maternel, mais seulement 3.5% connaissaient l'allaitement maternel exclusif pendant une certaine période de temps comme option d'alimentation du nourrisson. Néanmoins, la majorité des femmes pensait que le fait de ne pas allaiter était une option culturellement inacceptable.

**Conclusion:**

Les femmes de cette enquête connaissaient les méthodes de PTME, mais avaient une perception négative de certains aspects des services de conseil et de dépistage et de la recom mandation de l'option d'alimentation du nourrisson consistant à ne pas allaiter. Les travailleurs de la santé devraient fournir des services adaptés aux patientes et des conseils sur l'alimentation du nourrisson basés sur les recommandations de l'OMS et culturellement acceptables.

## Introduction

### Key focus

Human immunodeficiency virus infection and AIDS are leading causes of morbidity and mortality amongst women and children, particularly in the sub-Saharan African countries where the prevalence of HIV is high. Globally, in 2009, 370 000 children under 15 years of age were newly infected by HIV and an estimated 42 000–60 000 pregnant women died as result of HIV. Over 90% of the paediatric infections occurred through mother to child infection. Twenty low and middle income countries, including Nigeria, account for the highest estimated numbers of pregnant women living with HIV. In contrast, the number of new HIV infections amongst children and HIV-related deaths in mothers and children is virtually zero in high-income countries as a result of access to timely preventive services.^1^

Nigeria has the second highest number of people living with HIV in the world after South Africa, with an estimated 2.98 million people living with HIV, which constitutes nine per cent of the global HIV burden.^2, 3^ Women are disproportionately more affected than men, accounting for 58% of the national HIV burden. The prevalence of HIV is highest amongst women aged between 20 and 29 years^3^ and this situation has implications for the transmission of HIV from mothers to children.

Various factors contribute to the high burden of paediatric HIV infection in Nigeria and other sub-Saharan African countries. These include the high prevalence of HIV infection amongst women of reproductive age, large populations of women, high birth rates, and lack of access to effective interventions aimed at preventing mother to child transmission of HIV.^4^ Infants born to HIV-positive mothers in developing countries are at a 30–45% risk of being infected during pregnancy, delivery and breastfeeding in the absence of intervention to prevent mother to child infection.^5^ However, the rates of Mother to Child Transmission (MTCT) of HIV have fallen to as low as 2% in the developed countries with the introduction of the Prevention of Mother to Child (PMTCT) programmes.^5^ The services provided include HIV counselling and testing (HCT) for HIV in antenatal clinics and maternity wards, antiretroviral drug therapy, comprehensive antenatal care and safer delivery practices, appropriate infant feeding, counselling and support.^6^ The Nigerian PMTCT programme commenced in July 2002 with the goals of providing effective PMTCT services for women in the reproductive age group in selected health facilities and generating information for the formulation of a national policy and implementation guidelines for a comprehensive PMTCT intervention in Nigeria. There are currently several hundred sites providing these services in the six geo-political zones of Nigeria, under the coordination of the Federal Ministry of Health.^6^

#### Background

Conventionally, antenatal care for pregnant women provides opportunities for health education and counselling on many pregnancy-related topics including HIV and PMTCT. This is also an opportune time to offer HIV testing within the health facility allowing pregnant women and even couples to know their HIV status. It serves as an entry point into PMTCT services and to establish connections into the antiretroviral programmes.^7^ However, the acceptance of PMTCT services varies from place to place, with differing levels of awareness amongst pregnant women regarding AIDS and HIV transmission and prevention amongst pregnant women. In Nigeria, the level of knowledge is comparatively higher amongst pregnant women residing in the urban areas^8, 9, 10^ than amongst women in the rural areas.^11^

#### Trends

Studies have shown that not all pregnant women are willing to be tested despite the availability of HCT. Perez et al. reported that the reasons women gave for their refusal were mainly related to the need to consult their husbands or partners before being tested.^12^ Ignorance and socio-cultural barriers also hinder maximal utilisation of the PMTCT services and the adoption of the infant feeding recommendations by women in Nigeria as well as other African countries.^10, 13, 14^ In a study conducted in Lagos, Nigeria, almost all the respondents were willing to undergo HIV testing in pregnancy, particularly if it would prevent the transmission of HIV of their babies.^9^ Okonkwo et al. reported that almost all the women approved of Voluntary Counseling and Testing (VCT), mainly because they were aware that VCT could reduce the risk of transmission of HIV to their babies.^10^ However, a major condition for testing was that the results would be kept confidential. Overall, the acceptance of VCT appears to depend on the understanding that VCT has proven benefits for the unborn child. Socio-cultural factors such as stigmatisation and discrimination against HIV-infected individuals were identified as barriers toward widespread acceptance of VCT in Nigeria.^9^

Despite the fact that over 60% of Nigerians reside in rural areas, most of the prior research on pregnant women's perception of PMTCT services has focused on respondents attending secondary and tertiary health facilities in urban areas.^9, 10, 15^ Few studies have assessed the opinions of women receiving antenatal care at the primary health care level in rural communities. Furthermore, pregnant women's views regarding the recommended infant feeding options for HIV infected mothers have hitherto been underexplored.

#### Objectives

This study therefore aimed to provide information about the knowledge and perceptions of pregnant women accessing care at this level in rural communities regarding PMTCT, the antenatal services rendered and recommended infant feeding options.

#### Contribution to field

An assessment of the clients’ perceptions of the services rendered and information provided is extremely important because it reveals an important aspect of the actual level of success of the programme, particular amongst previously understudied rural communities. The findings obtained from this study would provide policy makers and managers with important information for improvement of the implementation of the PMTCT programme where required.

## Ethical Considerations

Ethical approval to conduct the study was obtained from the Oyo State Ministry of Health Ethical Review Committee. Permission to conduct the study was also obtained from the primary health care coordinator of the local government area and the health worker in charge of the health centre. Informed consent was obtained from each of the respondents before administering the questionnaire.

## Method

### Materials

The study population consisted of pregnant women attending the weekly antenatal care clinics in 2010 who consented to participate in the study. They were interviewed by four female research assistants. The total sample recruited to participate in the study consisted of all consenting pregnant women who presented at the antenatal care clinics during the two-month period of data collection. Pregnant women who did not consent to participate and those were seriously ill were excluded from the study.

### Setting

The study was conducted in Oluyole Local Government Area (OLGA), which is one of the 33 local government areas of Oyo State, in south-west Nigeria. The LGA is predominantly rural and has a population of 209 212 people, who are mainly of Yoruba ethnicity, engaged mainly in farming, petty trading and semi-skilled manual occupations. The respondents were interviewed at Olomi PHC centre, which is the largest PHC centre in Oluyole Local Government Area. This is the only primary health care facility offering comprehensive health care, including antenatal, delivery and postnatal services. The facility provides both outpatient and in-patient primary health care services for adults and children and is run by nurses and community health workers.

### Design

This was a descriptive survey of pregnant women. The required minimum sample size of the respondents was estimated using the equation for population surveys: *N = Z^2^ (100-p) p/d^2^*, where *Z* is a standard normal deviation usually set at 1.96. The confidence level was specified as 95% and the tolerable error margin (*d*) was 5%. The *p* was the estimated proportion of respondents with the outcome attribute, which was the percentage of women aware of MTCT (68%), obtained as from a previous study conducted by Abiodun.^8^ The minimum sample size obtained was 334. A total sample of pregnant women who presented at the weekly antenatal clinic which was held on Wednesdays and Thursdays during a two-month period in 2010 were interviewed by four female research assistants with a minimum of post secondary school diploma education. Four hundred and thirty pregnant women present at the antenatal clinic during the study period were approached; 400 of them gave consent and were interviewed; 12 refused to participate in the study, whilst 18 did not respond fully to the questionnaire, giving a response rate of 93%.

### Procedure

The respondents were interviewed using a semi-structured interviewer-administered questionnaire to obtain information on socio-demographic characteristics, previous delivery history, knowledge of modes of MTCT transmission of HIV and knowledge about PMTCT services. Information was also sought on the respondents’ knowledge and perceptions regarding the recommended infant feeding options for HIV-positive mothers and their utilisation of HIV counselling and testing. The interviews were conducted in a secluded location in the antenatal clinic of the PHC centre. The questionnaire was administered in Yoruba, which is the predominant language spoken by the residents in the study location.

### Statistical analysis

Completed questionnaires were inspected daily to check for completion, and to detect and correct errors. Data were entered and analysed with SPSS version 16. The occupational groups of the respondents were assessed using the Modified British Registrar General Social Classification.^16^ Professionals included managerial, teaching and other professional occupations; intermediate occupations included civil service occupations; skilled non-manual was made up of hairdressing, tailoring, et cetera; skilled manual referred to carpentry, welding, et cetera; unskilled workers included market porters and farmers. Those who were not currently working or were students were classified as unemployed. The Chi-square was used to test associations and the level of significance was set at *p*-value < 0.05.

## Results

The socio-demographic characteristics of the respondents are shown (Table 1). Their mean age was 27.3 ± 5.6 years, 224 (55.9%) were aged 25–34 years and almost all were of Yoruba ethnicity. Most of the respondents (262 or 65.3%) had completed secondary education. Almost all (98.7%) were currently employed and 225 (56.2%) were engaged in skilled non-manual occupations such as hairdressing and dressmaking. Regarding their pregnancy history, 279 (68.8%) had been pregnant before and 275 (68.6%) were in the third trimester of pregnancy.

**TABLE 1:** Socio-demographic characteristics of respondents *N* = 400.

Characteristics	*N*	%
**Age group (in years)**		
15–24	125	31.4
25–34	224	55.9
35–44	51	12.7
**Marital Status**		
Single	16	4
Cohabiting	48	14.5
Married	326	81.5
**Religion**		
Christianity	130	32.4
Islam	270	67.6
**Educational Attainment**		
Primary	95	23.8
Secondary	262	65.5
Tertiary Education	43	10.8
**Respondents’ occupation**		
Professional	32	8
Skilled manual	129	32.2
Skilled non-manual	225	56.2
Unemployed/Student	14	3.5
**Husband's occupation**		
Professional	60	15
Skilled manual	85	19.2
Skilled non-manual	77	21.2
Unskilled	139	34.8
Unemployed/student	41	9.7
**Ethnicity**		
Yoruba	396	99
Other †	4	1

*n*, Given as number of respondents.

† Others include Edo, Igbo and Beninoise.

All the respondents had heard of HIV and the media was the most commonly reported source of information about HIV (67.6%), followed by the health workers in the antenatal clinic (25.2%). Most respondents could correctly identify the modes of transmission of HIV, 366 (91.3%) identified sharing of sharp objects, and 352 (87.8%) knew about unsafe blood transfusions. However, mosquito bites, sharing of cutlery and eating utensils, and kissing were wrongly mentioned as modes of transmission by 215 (53.6%), 172 (42.9%), and 154 (38.4%) respectively.

### Respondents’ knowledge of mother to child transmission of HIV and its modes of prevention

Two thousand three hundred and six of the respondents (76.0%) knew that HIV infection could be transmitted from the mother to the child (Table 2). The commonest routes of transmission known were: through the placenta (277 or 69.3%), through breastfeeding (276 or 69.0%); and during delivery (272 or 68.0%). The methods of preventing mother to child transmission of HIV known to the respondents were giving ART to the mother during pregnancy (300 or 75.0%) and during labour (250 or 62.5%), giving ART to babies of HIV-positive women at birth (263 or 65.8%) and not breastfeeding the baby at all (247 or 61.8%).

**TABLE 2:** Respondents’ knowledge of routes of mother to child transmission of Human Immunodeficiency Virus and mode of prevention *N* = 400.

Variable	Yes		No		Don't know	
		
	*n*	%	*n*	%	*n*	%
**Route of MTCT**						
Through placenta	277	69.3	17	4.3	106	26.3
Through breastfeeding	276	69	22	5.5	112	25.3
During delivery	272	68	21	5.3	107	26.8
Through contact with the baby	80	20	219	54.8	111	25.3
**Prevention of MTCT**						
ART in pregnancy	300	75	13	3.3	87	21.8
ART to newborn	263	65.8	37	9.3	100	25.1
ART in labour	250	62.5	46	11.5	114	26.1
Not breastfeeding	247	61.8	54	13.5	99	24.8
Delivery by C-section	195	48.8	77	19.3	128	32.1

*n*, Given as number of respondents; Art, Antiretroviral therapy.

#### HIV counselling and testing

Two hundred and eighty-two (70.5%) of the respondents had received HIV counselling and testing (HCT), 84.0% and 15.0% had had the test conducted in the PHC antenatal clinic and secondary health facilities respectively. Most (385 or 96.3%) reported that that their husbands would approve of the test but 291 (72.8%) indicated that they had had to obtain permission from their husbands orpartners to undertake the test. Amongst those who had received the test, 262 (92.9%) reported that there was enough privacy, 260 (92.2%) understood the information, and 261 (92.6%) felt that nurses’ approach was acceptable but 96 (34.0%) felt that the test was forced on them. Uptake of HCT was significantly higher amongst women who were married (74.2%) compared with those who were single (50.0%) or cohabiting (55.2%), *p* = 0.003. The proportion of women who had received HCT increased significantly from 50.0% to 74.9% amongst those in their first and third semesters of pregnancy respectively (Table 3 and Table 4).

**TABLE 3:** Characteristics of respondents and previous Human Immunodeficiency Virus counselling and testing.

Socio-demographic Characteristics	Yes		No		*p*-value
	
	*N*	%	*n*	%	
**Age group (Years)**					
15–24	83	66.4	42	33.6	0.44
25–34	161	71.9	63	28.1	
≥35	38	74.5	13	25.5	
**Marital Status**					
Never married	8	50	8	50	0.003*
Cohabiting	32	55.2	26	44.8	
Married	242	74.2	84	25.8	
**Level of Education**					
Primary	65	68.4	30	31.6	0.5
Secondary	189	72.1	73	27.9	
Tertiary	282	65.1	15	34.9	
**Trimester of Pregnancy**					
First	6	50	6	50	0.01*
Second	70	61.9	43	38.1	
Third	206	74.9	69	25.1	

*n*, Given as number of respondents.

*p*-value is significant.

**TABLE 4:** Respondents’ assessment of prevention of mother to child transmission Services *N* = 282.

Variables	Yes		No		Don't know	
		
	*n*	%	*n*	%	*n*	%
There was enough privacy	262	92.9	19	6.8	1	0.3
The atmosphere was relaxed	261	92.6	20	7.1	1	0.3
The nurse's approach was acceptable	261	92.6	1	0.4	20	7.0
The information was well understood	260	92.2	2	0.7	20	7.1
Questions were well understood	260	92.2	20	7.1	2	0.7
The timing was appropriate	253	9.7.7	27	9.6	2	0.7
It was forced on me	96	34.0	166	8.9	20	7.0

*N*, Given as number of respondents.

Concerning the recommended infant feeding options for HIV-positive mothers, most (79.6%) mentioned infant formula, but only 14 (3.5%) were aware of exclusive breastfeeding for the first three to six months of the infant's life, and only 20 (5.0%) were aware of heating expressed breast milk. When the women were asked what infant feeding option they would select if they were HIV-positive, 303 (75.6%) said they would opt for formula feeding whilst 33 (8.2%) said they would breastfeed, and 64 (16.2%) were unsure of what their choice would be.

#### Respondents’ perception of non-breastfeeding mothers

On further probing regarding their perception of the non-breastfeeding or formula feeding option, 159 (39.7%) thought that the baby may not be healthy, 124 (31.2%) reported that they would not feel like a mother, 62 (15.5%) felt that people would speak negatively of them and 31 (7.7%) felt that the baby would not be well nourished and would be predisposed to premature death. With regards to their perception of a non-breastfeeding mother, 218 (54.4%) would guess that such mothers were HIV-positive, 87 (21.7%) would consider the mother to be irresponsible, whilst 43 (10.7%) would feel the mother may be showing off, 29 (7.2%), and 23 (5.7%) would believe that she is sick or mentally deranged, respectively.

## Discussion

This descriptive study documents the knowledge and perceptions of pregnant women utilising antenatal care services at the primary health care level regarding MTCT, the services rendered and recommended infant feeding options. Knowledge of MTCT was quite high, with most of the pregnant women being aware of how HIV could be transmitted from a mother to a child and that the transmission could be prevented through the use of ART in pregnancy and during labour, and about administration of ART to the newborn baby. Slightly over 70% of the women had undergone HCT, mainly at the antenatal clinic of the PHC facility where the study was conducted. This is lower than the uptake rate earlier reported for mothers attending a secondary health facility in Ibadan, the capital of Oyo State, in which 96.8% had received HCT at the ANC.^17^ The difference could be attributed to the higher level of implementation of PMTCT services at the secondary health care facility, which is one of the PEPFAR/APIN PMTCT sites in Oyo State. However, this rate is a marked improvement on the previous reports by Ekanem and Gbadegesin from a study conducted in 2005, which reported that only 39.2% of the study group had undergone the HIV test.^9^ This may be related to the scaling up of the PMTCT services in the country, especially at the PHC level, by the Federal Ministry of Health, national and international non-governmental organisations.

The assessment of the clients’ perception of the services rendered revealed that nine out of ten of the respondents were satisfied with the timing and the atmosphere in which the HCT test was conducted, but a third felt that the test was forced on them. This finding may be due to the fact that the nurses were overburdened by the number of clients and additional tasks required to render the PMTCT services. Similar findings were reported in a study conducted by Leshabari et al. in Tanzania, where counsellors felt overwhelmed by the constantly increasing workload pressure, resulting in compromise of the quality of work, and in some instances became aggressive when they were tired and emotionally distressed.^14^ However, it is in contrast to the report of a similar study conducted in Ibadan, which reported that the majority of the pregnant women visiting a secondary health facility were satisfied with services provided by the nurses.^17^

The need to obtain spousal approval to undertake HIV counselling and testing was clearly highlighted in this study. This finding is consistent with the report from a Ugandan study that women had to consult their husbands before having an HIV test.^18^ This is unsurprising in the African setting, where husbands or partners still play a critical role in decision making regarding their partners’ access to and utilisation of health care services. It further underscores the importance of involving men in PMTCT services.

The most commonly known infant feeding option for infants of HIV-positive mothers was formula feeding. However, most of the pregnant women had negative attitudes towards the non-breastfeeding option. Many of the women were of the opinion that fully formula-fed babies would be unhealthy, and the mothers would not feel like real mothers. Negative perceptions of mothers who refused to breastfeed their babies were also identified. These findings are similar to those obtained in another study conducted amongst pregnant women utilising a secondary health facility in Oyo State^17^ and are indicative of deep-seated socio-cultural values that should not be dismissed but rather be taken into consideration for the development of culturally relevant interventions. Apparently, the infant feeding information provided to these women has not incorporated the current WHO recommendations which support exclusive breastfeeding for six months unless formula feeding is acceptable, feasible, affordable, sustainable and safe.^19^ These recommendations have been informed by the findings of studies which have shown exclusive breastfeeding for six months is associated with decreased risk of HIV transmission compared to non-exclusive breastfeeding.^20, 21^

### Practical implications

Though pregnant women receiving antenatal care services at this rural PHC centre have access to PMTCT services, the health workers’ attitudes during service delivery may be hindering maximal adoption of HCT services. A key factor identified for acceptance of HCT is approval from the women's partners. The formula feeding option known to the majority of the women is not socio-culturally acceptable; consequently HIV-positive mothers adopting this infant feeding option run the risk of labelling, stigmatisation and discrimination. These women would probably not receive the required social support to comply with this recommendation and may resort to mixed feeding, with the attendant risk of MTCT of HIV and diarrhoeal disease in their infants.

### Limitations of the study

The main limitation of this study was that it was conducted in only one primary health facility and this may limit the extent to which the results can be generalised. Only women who attended the antenatal clinic and consented to participate in the study over the two-month study period were interviewed. This may have resulted in selection bias, because the views of women who utilised other sources of antenatal care such as private clinics and traditional birth attendants would not have been reflected in this study. However, the health facility was the only place in the local government area where PMTCT services were being provided at that time. Thus, only pregnant women visiting the facility would have been eligible to assess the PMTCT services delivered in that area. The population is fairly homogeneous and the cost of the services at the PHC facility is relatively low. Consequently, cost and differences in socio-economic status were probably not the reasons for utilisation of these services by the respondents but rather confidence in the care provided and accessibility. Therefore, it could safely be assumed that the results obtained were applicable to the women in this area who utilise public health care facilities.

Perceptions about current infant feeding recommendations were only obtained from the respondents visiting the clinic. Future research should involve women attending antenatal clinics in primary health care clinics in several locations. Similar studies should also be conducted amongst males and community members to assess their perceptions regarding HIV, PMTCT and infant feeding recommendations.

### Recommendations

Efforts should be made to ensure maximum access to PMTCT services by pregnant women attending PHC clinics in rural areas. Health workers providing PMTCT services require update training on various aspects of the programme, including the current recommendations for infant feeding. Strategies to promote male involvement need to be explored. The infant feeding counselling should take into cognisance the current WHO infant feeding recommendations.

## Conclusion

Although about 70% of the women who were interviewed in this study had received HIV counselling and testing, it is important to focus on the fact that 30% had not received any counselling. Adding to the concern in this rural facility is the fact that for about 70% of the women this was not their first pregnancy, and again about 70% were already in their third trimester of pregnancy. All these factors limits the opportunity to prevent successful prevention of mother to child transmission and emphasise the importance to perform further studies to alert policy makers on shortcomings.

Most of the women in this study had received HCT, but had negative perceptions about certain aspects of the services and the infant feeding recommendations. These issues as well as cultural perceptions need to be closely investigated. Although the study has some limitations, it remains important to address these issues to ensure optimal utilisation of the PMTCT services. Dissemination of scientifically sound and culturally acceptable information on PMTCT services, including infant feeding options, is of utmost importance.
